# Roles of the EnvZ/OmpR Two-Component System and Porins in Iron Acquisition in *Escherichia coli*

**DOI:** 10.1128/mBio.01192-20

**Published:** 2020-06-23

**Authors:** Henri Gerken, Phu Vuong, Ketaki Soparkar, Rajeev Misra

**Affiliations:** a School of Life Sciences, Arizona State University, Tempe, Arizona, USA; Emory University School of Medicine

**Keywords:** iron homeostasis, two-component signal transduction systems, porins, ferric transport, ferrous transport, EnvZ/OmpR

## Abstract

Escherichia coli secretes high-affinity Fe^3+^ chelators to solubilize and transport chelated Fe^3+^ via specific outer membrane receptors. In microaerobic and anaerobic growth environments, where the reduced Fe^2+^ form is predominant, ferrous transport systems fulfill the bacterial need for iron. Expression of genes coding for iron metabolism is controlled by Fur, which when bound to Fe^2+^ acts as a repressor. Work carried out here shows that the constitutively activated EnvZ/OmpR two-component system, which normally controls expression of the *ompC* and *ompF* porin genes, dramatically increases the intracellular pool of accessible iron, as determined by whole-cell electron paramagnetic resonance spectroscopy, by inducing the OmpC/FeoB-mediated ferrous transport pathway. Elevated levels of intracellular iron in turn activated Fur, which inhibited the ferric transport pathway but not the ferrous transport pathway. The data show that the positive effect of constitutively activated EnvZ/OmpR on *feoB* expression is sufficient to overcome the negative effect of activated Fur on *feoB*. In a *tonB* mutant, which lacks functional ferric transport systems, deletion of *ompR* severely impairs growth on rich medium not supplemented with iron, while the simultaneous deletion of *ompC* and *ompF* is not viable. These data, together with the observation of derepression of the Fur regulon in an OmpC mutant, show that the porins play an important role in iron homeostasis. The work presented here also resolves a long-standing paradoxical observation of the effect of certain mutant *envZ* alleles on iron regulon.

## Abstract (precis)

The work presented here solved a long-standing paradox of the negative effects of certain missense alleles of *envZ*, which codes for kinase of the EnvZ/OmpR two-component system, on the expression of ferric uptake genes. The data revealed that the constitutive *envZ* alleles activate the Feo- and OmpC-mediated ferrous uptake pathway to flood the cytoplasm with accessible ferrous iron. This activates the ferric uptake regulator, Fur, which inhibits ferric uptake system but cannot inhibit the *feo* operon due to the positive effect of activated EnvZ/OmpR. The data also revealed the importance of porins in iron homeostasis.

## INTRODUCTION

Iron, used as a redox center by many enzymes, is an essential trace metal required by almost all living organisms. The intracellular level of free catalytically active iron is typically kept low due to its toxic effects. Free ferrous iron reacts with hydrogen peroxide, a natural by-product of aerobic respiration, to generate highly toxic hydroxyl radicals (OH**^⋅^**) via the Fenton reaction (1). Due to this potentially damaging property of iron, there exists an intricate balance between iron transport, utilization, and storage. Most bacteria possess mechanisms to import iron in its oxidized ferric state (Fe^3+^), reduced ferrous state (Fe^2+^), or both (for reviews, see references 2 and 3). The solubility of these two iron forms differs drastically at neutral pH: ferric iron has extremely low solubility at 10^−18^ M, whereas ferrous iron is readily soluble at 10^−1^ M. To take up ferric iron, bacteria have developed high-affinity ferric iron chelators called siderophores to capture, solubilize, and deliver insoluble iron into the cell (4). Unlike the Fe^3+^ transport system, which requires a number of proteins involved in siderophore synthesis and Fe^3+^ siderophore acquisition, the Fe^2+^ transport system appears to consist of mainly one protein, FeoB (5). The FeoB protein is synthesized from the *feoABC* operon, whose expression is activated by Fnr, an anaerobic transcriptional regulator (5). FeoB is a highly conserved, 773-residue inner membrane protein that contains several GTP-binding motifs (6–8). In the absence of FeoB or FeoA, Fe^2+^ uptake is either virtually abolished (Δ*feoB*) or mildly reduced (Δ*feoA*) (5). The function of FeoC, which is present only in members of the *Enterobacteriaceae* family, is unknown (7, 8). FeoB and its homologs are required for full virulence in many bacteria, including Escherichia coli (9), Salmonella enterica serovar Typhimurium (10, 11), and Helicobacter pylori (12).

Fur (ferric uptake regulator) in E. coli and its orthologs in many Gram-negative and Gram-positive bacteria are the master regulator of genes encoding both ferric and ferrous iron acquisition functions, as well as siderophore synthesis and uptake (13, 14). Cells lacking Fur experience iron overload that causes oxidative damage and mutagenesis (15). Fur-regulated genes contain one or more Fur-binding sites around the −35 and −10 regions of the promoter, often referred to as the Fur boxes (16, 17). Fur uses Fe^2+^ as a cofactor: when the level of available Fe^2+^ increases in the cell, it binds to Fur and enhances its affinity for DNA by almost 1,000-fold (2). The active Fur-Fe^2+^ complex then binds to a Fur box and represses transcription of the iron acquisition gene. RyhB is a small regulatory RNA whose transcription is also repressed by Fur-Fe^2+^ (18). Consequently, when Fur is active, the levels of RyhB are low, resulting in stabilization and translation of over a dozen mRNAs encoding nonessential iron utilization proteins, including those that store iron (Bfr), detoxify superoxide (SodB), and catalyze steps of the tricarboxylic acid cycle (AcnA and SdhCDAB) (19). Thus, excess Fe^2+^ activates Fur to halt further iron uptake and at the same time, promotes the utilization of Fe^2+^, and inversely, low intracellular iron level induces iron uptake and utilization (20). Recent genome-wide analyses revealed a more comprehensive profile of Fur and RyhB regulons (21, 22).

Whereas Fur and RyhB are the principal determinants of iron homeostasis in E. coli, evidence exists supporting the involvement of some two-component signal transduction systems (TCS) in iron homeostasis. EnvZ and OmpR are the archetypal TCS in E. coli, where EnvZ serves as a sensor kinase and OmpR as a response regulator (23). They respond to medium osmolarity and influence the expression of OmpC and OmpF, the two major porins that facilitate the diffusion of small hydrophilic solutes (∼600 Da) across the outer membrane (24). OmpC is preferentially expressed in high osmolarity, whereas OmpF expression is favored in low osmolarity (25). Microarray data from an Δ*ompR* Δ*envZ* background showed a significant increase in the expression of a number of Fur-regulated genes, particularly those involved in enterobactin siderophore synthesis and transport (26). Over 3 decades ago, Lundrigan and Earhart (27) reported that in a *perA* (*envZ*) mutant background, the levels of three iron-regulated proteins were significantly reduced. These authors suggested that this could be due to a posttranscriptional defect. Later, it was speculated that this inhibition could be due to the indirect effects of *envZ*/*ompR*, leading to alterations in the structure and diffusion properties of the outer membrane (28). While characterizing revertants of an E. coli mutant defective in outer membrane biogenesis, we discovered several pleiotropic *envZ* alleles conferring an OmpC^+^ OmpF^−^ LamB^−^ phenotype (29). These alleles were hypothesized to biochemically lock EnvZ into a conformation that causes increased OmpR phosphorylation. This activated EnvZ/OmpR state is thought to enable OmpR to bind to promoters with weak OmpR-binding affinities. One such pleiotropic *envZ* allele, *envZ_R397L_*, was characterized in detail (29). The preliminary whole genomic microarray analysis of the *envZ_R397L_* mutant carried out in our laboratory found that the largest group of genes (>50) affected by the activated EnvZ_R397L_/OmpR^+^ background belonged to the Fur regulon (30; unpublished data).

In this study, we show that EnvZ_R397L_ exerts its effect on the Fur regulon in part by increasing the accessible intracellular pool of iron via the OmpC-FeoB-mediated Fe^2+^ transport pathway. This, in turn, activates Fur and downregulates the Fe^3+^ transport pathway. Our analyses also revealed the critical roles of EnvZ/OmpR and porins in iron homeostasis in the Δ*tonB* background where high-affinity iron transport systems are nonfunctional.

## RESULTS

### Effects of *envZ_R397L_* on the ferric transport system.

**FIG 1:**
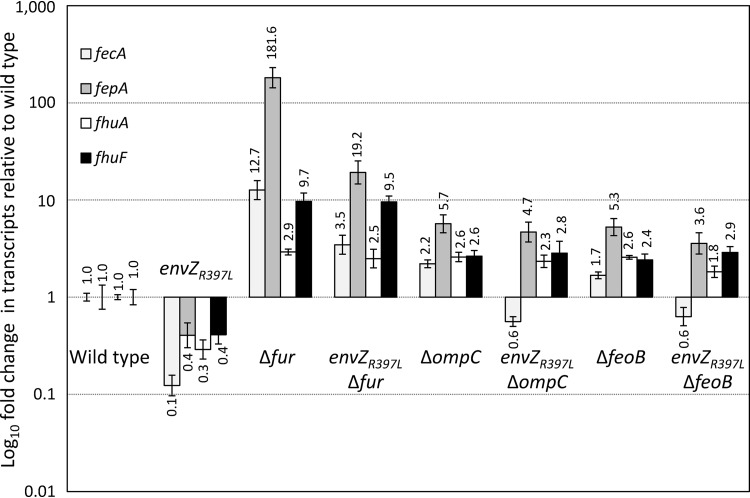
Determination of *fecA*, *fepA fhuA*, and *fhuF* expression in different genetic backgrounds by real-time quantitative PCR (RT-qPCR). RNA was isolated from bacterial cultures grown to mid-log phase. Relative quantification of transcripts was performed using the 2^–ΔΔ^*^CT^* method, with *ftsL* and *purC* serving as the reference genes. The relative fold changes in gene expression and error bars representing standard deviations are shown. The bacterial strains used included RAM1292 (wild type), RAM1541 (*envZ_R397L_*), RAM2697 (Δ*fur*), RAM2698 (*envZ_R397L_* Δ*fur*), RAM2699 (Δ*ompC*), RAM2700 (*envZ_R397L_* Δ*ompC*), RAM2701 (Δ*feoB*), and RAM2702 (*envZ_R397L_* Δ*feoB*).

We first set out to investigate the effects of *envZ_R397L_* on the Fur regulon. RNA isolated from mid-log-phase grown cells was converted to cDNA, and the levels of various transcripts were analyzed by real-time quantitative PCR (RT-qPCR). The data in Fig. 1 show relative transcript levels of four Fur-regulated genes: *fecA*, *fepA*, *fhuA*, and *fhuF*. In the *envZ_R397L_* background, their transcript levels went down 10 (*fecA*)-, 3 (*fhuA*)-, and 2.5 (*fepA* and *fhuF*)-fold relative to the wild-type (EnvZ^+^) strain. As expected, in a Δ*fur* background their expression was derepressed, resulting in a dramatic increase in their transcripts (Fig. 1). In that background, the presence of *envZ_R397L_* was still able to reduce *fecA* and *fepA* transcript levels 3.6- and 9.5-fold, respectively, but not that of *fhuA* and *fhuF*, which experienced a <20% reduction (Fig. 1). Using the chromosomally integrated *fepA*::*lacZ* and *fhuA*::*lacZ* gene fusion constructs, we were able to recapitulate the key RNA data shown in Fig. 1 (see Fig. S1 in the supplemental material). This indicated that EnvZ_R397L_/OmpR or factors under the activated TCS control could also downregulate *fecA* and *fepA* transcription in the absence of Fur. In contrast, the negative effect of *envZ_R397L_* on *fhuA* and *fhuF* expression requires Fur. Moreover, the repressive effect of *envZ_R397L_* on *fecA* and *fepA* in the *fur*^+^ background was found to be independent of OmrA and OmrB (Fig. S2), the two EnvZ/OmpR-dependent small regulatory RNAs whose overexpression from plasmids was previously shown to downregulate *fecA*, *fepA*, and other Fur-regulated genes (31). It is worth mentioning that the *envZ_R397L_* allele has been previously shown to increase *omr*::*lacZ* expression almost 10-fold (29).

10.1128/mBio.01192-20.1 FIG S1 Determination of *fepA*, *fhuA*, and *feo* expression using chromosomally integrated *lacZ* fusions. β-Galactosidase activities (shown in Miller units) were measured from at three independent biological replicates in various genetic backgrounds as shown. Error bars represent standard deviations. Download FIG S1, TIF file, 0.6 MB. Copyright © 2020 Gerken et al. 2020 Gerken et al. This content is distributed under the terms of the Creative Commons Attribution 4.0 International license.

10.1128/mBio.01192-20.2 FIG S2 Effects of OmrA and OmrB deletion on *fecA* and *fepA* in wild-type and EnvZ_R397L_ backgrounds. Determination of *fecA* and *fepA* expression was carried out using RT-qPCR. Data were obtained from two independent biological samples and two technical replicates. Error bars represent standard deviations. Download FIG S2, TIF file, 0.2 MB. Copyright © 2020 Gerken et al. 2020 Gerken et al. This content is distributed under the terms of the Creative Commons Attribution 4.0 International license.

Expression of OmpC is activated constitutively in the *envZ_R397L_* background, while that of OmpF and LamB is severely inhibited (29). To determine whether OmpC is somehow involved in the *envZ_R397L_*-mediated downregulation of *fecA*, *fepA*, *fhuA*, and *fhuF*, we examined their transcript levels in the Δ*ompC* and Δ*ompC envZ_R397L_* backgrounds. Remarkably, without OmpC, *envZ_R397L_* was unable to exert any significantly negative effect on *fepA*, *fhuA*, and *fhuF* expression, while the effect on *fecA* diminished from 10-fold in the presence of OmpC to <2-fold without OmpC (Fig. 1). Interestingly, the levels of all four transcripts went up in Δ*ompC* cells (Fig. 1). We theorize that without OmpC, diffusion of Fe^2+^ into the cell is decreased and the less active Fur fails to fully repress *fecA*, *fepA*, *fhuA*, and *fhuF* expression.

If the intake of Fe^2+^ by OmpC porin increases active Fur-Fe^2+^ levels, then the absence of FeoB, the Fe^2+^-specific iron transporter, should also interfere with this activation and abrogate the Fur-mediated effects of *envZ_R397L_* on *fecA*, *fepA*, *fhuA*, and *fhuF*. Indeed, just like in the Δ*ompC* background, *fecA*, *fepA*, *fhuA*, and *fhuF* transcript levels went up in the Δ*feoB* background, and *envZ_R397L_* could either no longer impose a significant negative effect (*fepA*, *fhuA*, and *fhuF*) or the effect was significantly reduced (*fecA*).

### Effects of *envZ_R397L_* on the ferrous transport system.

The data presented in Fig. 1 show the involvement of the FeoB ferrous iron transporter and OmpC porin in *envZ_R397L_*-mediated downregulation of the ferric iron transport system. While *ompC* expression increases in the *envZ_R397L_* background (29), the status of the *feo* operon in this background is unknown. The *feo* operon is under the negative control of Fur (5). Consequently, if higher Fur-Fe^2+^ activity is present in the *envZ_R397L_* background, as we have suggested above, then the expression of the *feo* operon, like that of *fecA*, *fepA*, *fhuA*, and *fhuF*, should also be inhibited. This, however, will be incongruent with our data showing *envZ_R397L_*’s dependence on *feoB* for its effects. We therefore hypothesized that *feo* expression, like that of *ompC*, is activated by *envZ_R397L_* to such a degree that it more than compensated for the *feo* downregulation by increased Fur-Fe^2+^ activity.

**FIG 2:**
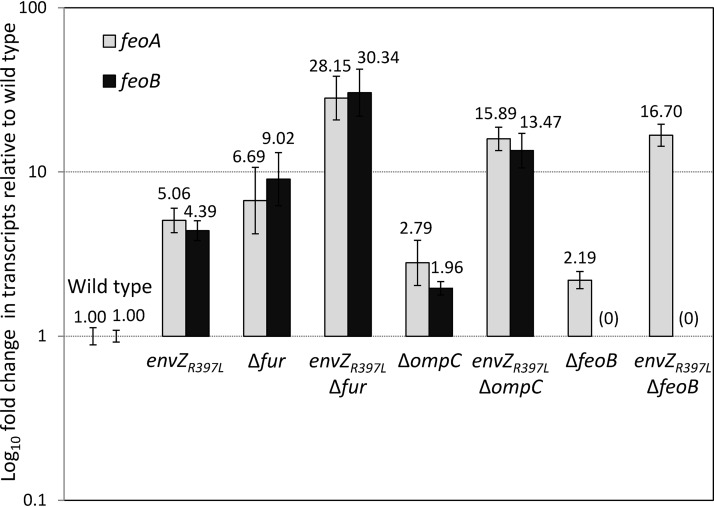
Determination of the relative gene expression of *feoA* and *feoB* by RT-qPCR. RNA was isolated from bacterial cultures grown to mid-log phase. Relative quantification of transcripts in various genetic backgrounds was performed using the 2^–ΔΔ^*^CT^* method, with *ftsL* and *purC* serving as reference genes. The relative fold changes in gene expression and error bars representing standard deviations are shown. The bacterial strains used included RAM1292 (wild type), RAM1541 (*envZ_R397L_*), RAM2697 (Δ*fur*), RAM2698 (*envZ_R397L_* Δ*fur*), RAM2699 (Δ*ompC*), RAM2700 (*envZ_R397L_* Δ*ompC*), RAM2701 (Δ*feoB*), and RAM2702 (*envZ_R397L_* Δ*feoB*).

To test these possibilities, we analyzed *feoA* and *feoB* transcript levels in different genetic backgrounds by RT-qPCR (Fig. 2). Note that *feoABC* are part of a contiguous operon and therefore likely expressed from a polycistronic message. Consequently, *feoA* and *feoB* transcript analysis probes their respective coding regions in a polycistronic message. In the EnvZ_R397L_ background, *feoA* and *feoB* transcript levels went up dramatically over those in the *envZ*^+^ control strain. As expected, their levels also went up in the Δ*fur* background. Interestingly, in the *envZ_R397L_* Δ*fur* background *feoA* and *feoB* transcript levels increased well above those in the individual mutation backgrounds, indicating that *envZ_R397L_* and Δ*fur* act independently and synergistically to enhance *feo* expression. Again, these observations were recapitulated using the chromosomally integrated *feo*::*lacZ* fusion (Fig. S1). These data support our hypothesis that *envZ_R397L_* activates *feo* expression in a fashion that counteracts repression by higher levels of Fur-Fe^2+^.

We then examined the effects of *envZ_R397L_* on *feoA* and *feoB* transcript levels in the absence of OmpC or FeoB. Without OmpC or FeoB, a modest 2-fold increase in *feoA* and *feoB* (Δ*ompC*) or *feoA* (Δ*feoB*) transcripts was observed (Fig. 2). We interpret this to reflect a modest relief in the Fur-mediated repression of the *feo* operon, since we have already implicated OmpC and FeoB in the ferrous iron transport and increase in Fur-Fe^2+^ levels (Fig. 1). The presence of *envZ_R397L_* in the Δ*ompC* or Δ*feoB* background led to an increase in *feoA* and *feoB*, or *feoA* transcripts, respectively, in a synergistic fashion, which is likely due to the simultaneous activation of *feo* expression by *envZ_R397L_* and a modest decrease in the Fur-mediated repression of *feo* from the absence of OmpC and FeoB. These data showed that *envZ_R397L_* inhibits ferric transport pathway but activates ferrous transport pathway.

### Intracellular iron levels in the *envZ_R497L_* mutant.

**FIG 3:**
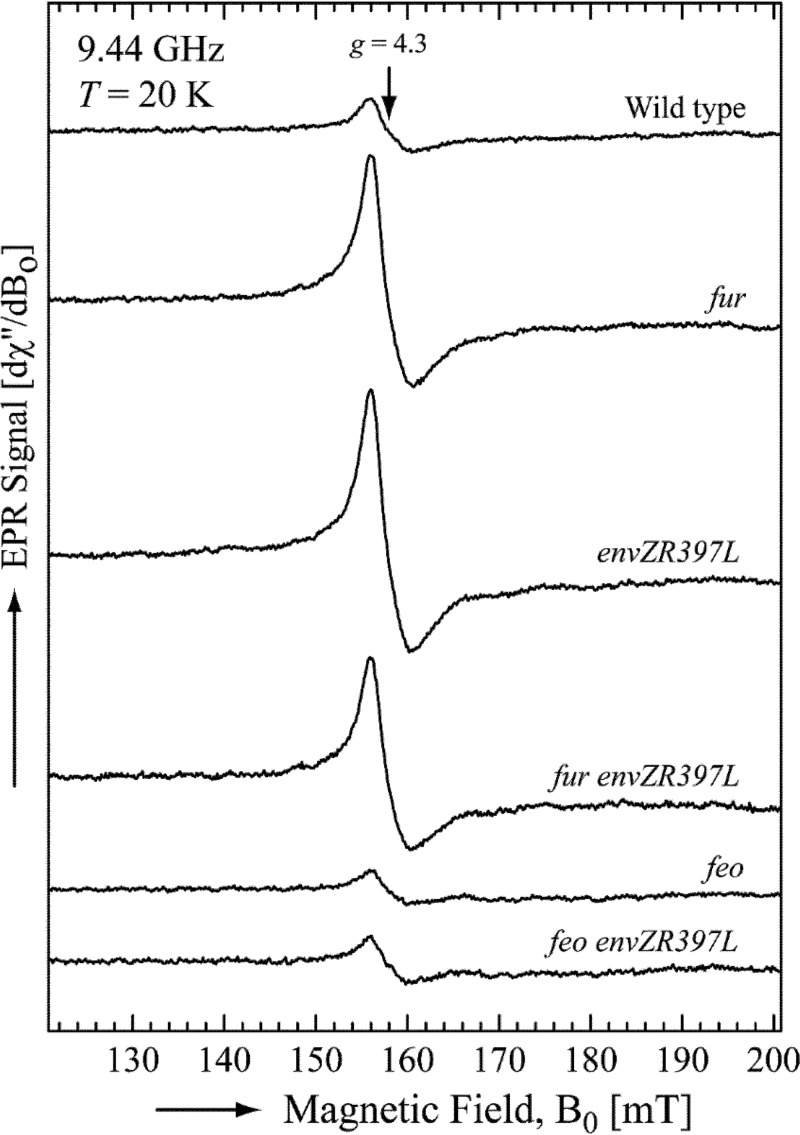
Determination of the intracellular free iron concentration. The averages of five ferric-chelate EPR scans per strain are shown. All scans were normalized to the final culture OD_600_ used in the measurements. The EPR parameters were as follows: microwave power, 10 mW; microwave frequency, 9.44 GHz; center field, 160 mT; sweep width, 80 mT; modulation amplitude, 1.25 mT; and modulation frequency, 100 kHz. The free intracellular iron concentrations, calculated as described in Materials and Methods, were as follows: wild type, 32 μM; Δ*fur*, 120 μM; *envZ_R397L_*, 135 μM; Δ*fur envZ_R397L_*, 105 μM; Δ*feoB*, 20 μM; and Δ*feoB envZ_R397L_*, 29 μM. The bacterial strains used included RAM1292 (wild type), RAM1541 (*envZ_R397L_*), RAM2697 (Δ*fur*), RAM2698 (*envZ_R397L_* Δ*fur*), RAM2701 (Δ*feoB*), and RAM2702 (*envZ_R397L_* Δ*feoB*).

The OmpC/Feo-mediated increase in Fur-Fe^2+^ activity in the *envZ_R397L_* background implies that the cytoplasm of the *envZ_R397L_* mutant contains higher levels of accessible iron than that in the cytoplasm of the EnvZ^+^ cell. To test this directly, we measured the intracellular pool of accessible iron by whole-cell electron paramagnetic resonance (EPR) spectroscopy, a method established in the Imlay laboratory (32). The data presented in Fig. 3 show that the wild-type (EnvZ^+^) strain had 32 μM of accessible intracellular iron. Expectedly, this level rose 4-fold to 120 μM in the Δ*fur* mutant. Remarkably, the level of accessible iron in the *envZ_R397L_* was also very high (135 μM) and remained high in the Δ*fur envZ_R397L_* double mutant (105 μM), thus supporting the notion that a higher pool of accessible iron in the *envZ_R397L_* background is responsible for the higher levels of active Fur-Fe^2+^.

Next, we tested whether the FeoB-mediated ferrous transport pathway is responsible for the elevated level of accessible iron in the *envZ_R397L_* mutant. The accessible iron level in the Δ*feoB* mutant was 20 μM or 35% less than the parental *feoB*^+^ strain (Fig. 3), explaining the observed deregulation of the Fur regulon in the Δ*feoB* mutant (Fig. 1 and 2). Strikingly, without *feoB*, *envZ_R397L_* failed to increase intracellular iron levels (Fig. 3), thus confirming the involvement of the FeoB-mediated ferrous transport in elevating the intracellular pool of iron, which, in turn, would increase Fur-Fe^2+^ levels and repress expression of *fecA*, *fepA*, *fhuA*, and *fhuF*. As described below, EnvZ/OmpR play a more direct role in activating *feo* expression to overcome the Fur-mediated downregulation.

### Effects of *envZ_R397L_* on *fepA* and *feo* requires phosphorylated OmpR.

Previously, it was shown that the pleiotropic effects of the mutant *envZ* allele, *envZ473* with its V241G substitution, is mediated through OmpR (33). In that study, the authors did not analyze the iron regulon. Here, we sought to test whether the effect of *envZ_R397L_* on iron regulon requires functional OmpR. We used a missense allele of *ompR* with a D55Y substitution, which confers a null phenotype with respect to *ompC* and *ompF* expression, presumably due to the inability of the mutant OmpR to be phosphorylated. The conserved D55 residue of OmpR is the site of phosphorylation (34). The *ompR_D55Y_* allele was isolated in a *fepA*::*lacZ envZ_R397L_* background among Lac^+^ revertants (R. Misra, unpublished data). Using a linked chloramphenicol resistance (Cm^r^) marker, we transduced the *ompR_D55Y_ envZ_R397L_* mutations into a *feo*::*lacZ* background so that the effects of the mutant *ompR* and *envZ* alleles on *feo* expression can be determined. It is worth noting that although *ompR*/*envZ* are highly linked to the *feo* operon, we were able to construct the above strain since *feo*::*lacZ* is marked by the kanamycin resistance (Km^r^) gene and the mutant *ompR*/*envZ* alleles produce a distinct porin phenotype.

**FIG 4:**
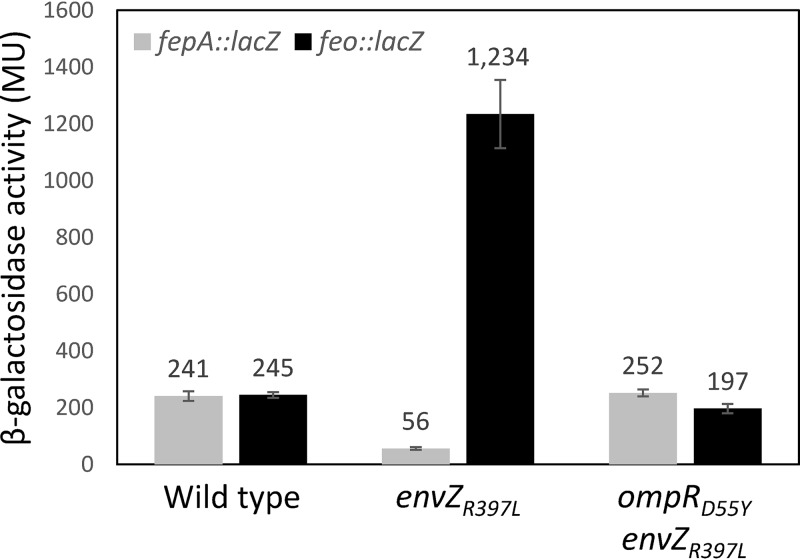
Determination of *fepA*::*lacZ* and *feo*::*lacZ* activities in various genetic backgrounds. The β-galactosidase activities were measured from two independent overnight grown cultures. Error bars represent standard deviations. The bacterial strains used included RAM2920 (*ompR*^+^ *envZ*^+^ *fepA*::*lacZ*), RAM2921 (*ompR*^+^ *envZ_R397L_ fepA*::*lacZ*), RAM2922 (*ompR_D55Y_ envZ_R397L_ fepA*::*lacZ*), RAM2923 (*ompR*^+^ *envZ*^+^ *feo*::*lacZ*), RAM2924 (*ompR*^+^ *envZ_R397L_ feo*::*lacZ*), and RAM2925 (*ompR_D55Y_ envZ_R397L_ feo*::*lacZ*).

Data presented in Fig. 4 show that *envZ_R397L_* reduced *fepA*::*lacZ* expression ∼4-fold, whereas *ompR_D55Y_* abolished this effect of *envZ_R397L_*. Likewise, the presence of *envZ_R397L_* elevated *feo*::*lacZ* expression 5-fold and again *ompR_D55Y_* abolished this increase in *feo* expression. Curiously, *feo*::*lacZ* expression in the *ompR_D55Y_ envZ_R397L_* background was slightly lower than that seen in the wild-type background, suggesting a role for functional OmpR in the expression of the *feo* operon. Together, these data show unambiguously that the negative and positive effects of *envZ_R397L_* on *fepA* and *feo*, respectively, require functional OmpR.

### Direct regulation of *feoABC* operon by EnvZ/OmpR.

**FIG 5:**
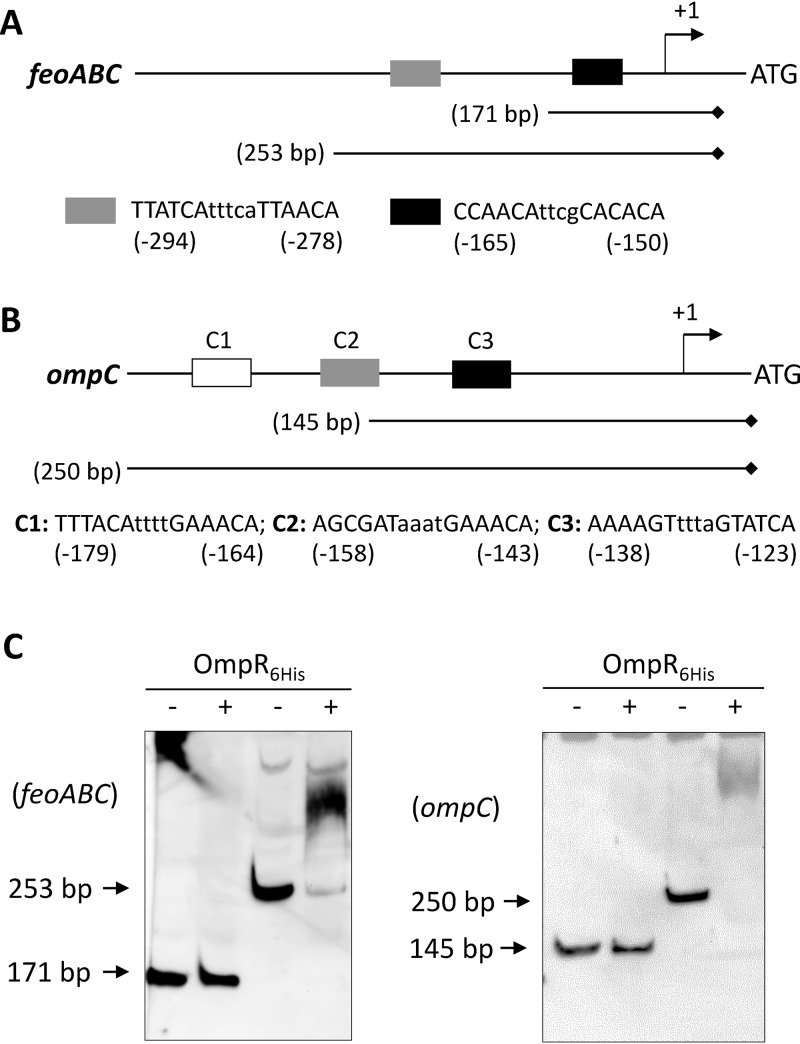
*In vitro* binding of purified OmpR_6His_ to the *feoABC* and *ompC* promoter regions. DNA binding was examined by EMSA using biotin-labeled DNA fragments of various lengths generated by PCR. (A) Diagram showing the regulatory region of the *feoABC* operon (not drawn to scale). Gray and black boxes represent possible OmpR binding sequences. Nucleotide numberings are relative to the *feoA* start codon. The relative positions and lengths of the two DNA fragments used in EMSA are shown. Diamond marks the biotin-labeled end of the DNA probe. (B) Diagram showing the regulatory region of the *ompC* gene (not drawn to scale). Three boxes represent the known OmpR binding sites; the DNA sequences of all three OmpR binding sites—C1, C2, and C3—are shown. Nucleotide numberings are relative the *ompC* start codon. The relative positions and lengths of the two DNA fragments used in the EMSA are shown. A diamond indicates the biotin-labeled end of the DNA probe. (C) Polyacrylamide gels showing EMSA results. Plus and minus signs denote the presence and absence, respectively, of OmpR in the reaction mixture prior to gel electrophoresis. Gels were electroblotted, and DNA bands were detected by treating membranes with stabilized streptavidin-HRP conjugate, followed by luminol/enhancer and stable peroxide. Arrows point to positions of unshifted DNA fragments.

The data in Fig. 2 and 4 showed a dramatic increase in the *feo* transcript/transcription levels in the *envZ_R397L_*/*ompR*^+^ background. This could be due to the direct regulation of *feo* by OmpR or an effect of an OmpR-controlled factor on the *feo* promoter or *feo* transcript. We took cues from an earlier publication that showed overexpression of RstA, the response regulator of the RstB/RstA TCS, upregulated *feoB* expression and repressed the Fur regulon in *Salmonella* Typhimurium (35). Electrophoretic mobility shift assays (EMSAs) showed direct binding of RstA to the *feo* promoter sequence (35). Moreover, these authors identified the “RstA motif” (TACA-N_6_-TACA) upstream of the *S.* Typhimurium *feoA* gene of the *feo* operon (35). Although OmpR recognition sequences are quite degenerate (36, 37), one of the motifs–GTTACANNNN–resembles that of RstA (Fig. 5A). Indeed, both RstA and OmpR regulate some of the same genes by binding to overlapping promoter sequences (38). Our initial assessment detected two potential sequences (−294)-TTATCAtttcaTTAACA-(–278) and (−165)-CCAACAttcgCACACA-(–150) upstream of the *feoA* ATG codon that might contain both RstA and OmpR binding motifs (Fig. 5A).

EMSA was carried out to test whether OmpR can bind directly to the *feo* promoter region. The coding region of *ompR* was cloned into an expression vector, pET24d(+). To aid in protein purification, six consecutive histidine codons were included at the 3′ end of the gene during cloning, and the protein was purified to near homogeneity by metal affinity chromatography (Fig. S3). The purified protein was used directly without *in vitro* phosphorylation. Using biotinylated primers, two DNA templates of the *feo* regulatory region, encompassing the predicted OmpR binding motifs, were amplified by PCR (Fig. 5A). As a positive control for OmpR binding, two *ompC* DNA fragments were also included for EMSA (Fig. 5B). No DNA gel shift occurred with the smaller *feoB* DNA fragment containing one of the predicted OmpR binding motifs (Fig. 5C). However, the larger *feo* promoter fragment, containing the upstream predicted OmpR binding motif, displayed shifts after incubation with purified OmpR_6His_ (Fig. 5C). Consistent with these *in vitro* data, we found that overexpression of OmpR_6His_ from a pBAD24 replicon increased *feo*::*lacZ* expression 2-fold (from 140 ± 8 Miller units in the pBAD24 vector containing strain to 296 ± 25 Miller units in the strain containing pBAD24-*ompR_6His_*). OmpR bound to the *ompC* promoter fragment containing the high-affinity OmpR-binding motif C1 (39), but not with the one containing the partial C2 and the entire C3 motif (Fig. 5C). Incidentally, only the *ompC* fragment, containing all three OmpR motifs, expressed the promoterless *lacZ* gene in an OmpR-dependent manner (Fig. S4), thus corroborating the EMSA data. Together, these data indicated that OmpR positively regulates *feo* expression by directly binding to the *feo* promoter region.

10.1128/mBio.01192-20.3 FIG S3 SDS-PAGE analysis of fractions containing OmpR_6His_ obtained after nickel affinity chromatography. Protein samples were visualized after Coomassie brilliant blue staining. Fractions shown in the box were pooled and used for DNA binding studies. The position of OmpR_6His_ is shown. Download FIG S3, TIF file, 0.9 MB. Copyright © 2020 Gerken et al. 2020 Gerken et al. This content is distributed under the terms of the Creative Commons Attribution 4.0 International license.

10.1128/mBio.01192-20.4 FIG S4 Determination of *ompC*::*lacZ* activities of *constructs* carrying two different lengths of the *ompC* promoter regions in front of a promoterless *lacZ* gene. *ompC145*::*lacZ* and *ompC250*::*lacZ* contain 145 and 250 bp, respectively, of the *ompC* promoter region, including ATG. The β-galactosidase activities in *ompR*^+^ or Δ*ompR* backgrounds were measured from two independent cultures grown to late log phase. Error bars represent standard deviations. Download FIG S4, TIF file, 0.8 MB. Copyright © 2020 Gerken et al. 2020 Gerken et al. This content is distributed under the terms of the Creative Commons Attribution 4.0 International license.

### Role of porins in iron homeostasis.

The data in Fig. 1 and 4 revealed a possible mechanism by which a pleiotropic allele of *envZ* downregulates the ferric transport systems by employing the OmpC/FeoB-mediated ferrous transport pathway. While these data implicated EnvZ/OmpR and OmpC in iron transport, the use of a pleiotropic *envZ* allele may have created an unnatural genetic environment in which EnvZ/OmpR and porins become involved in iron homeostasis. To eliminate this possibility, we determined the roles EnvZ/OmpR and porins in iron transport using the null alleles of *ompR* and the porin genes. Before testing their roles, we disabled the high-affinity ferric transport system, since porins likely mediate iron transport by simple diffusion of ferrous or small iron-chelated compounds, and this passive activity of porins will likely be masked by the high-affinity iron transport system. In E. coli, the high-affinity iron transport principally involves a ferric chelator, enterobactin, and TonB that interacts with the outer membrane iron receptors for the release of chelator-Fe^3+^ complexes bound to the receptor. Consequently, we disabled the ferric iron transport by deleting *aroB*, *tonB* or both. The *aroB* gene encodes 3-dehydroquinate synthase, which is required for the second step of the chorismate pathway in the synthesis of enterobactin, aromatic amino acids, and other important compounds (40).

**FIG 6:**
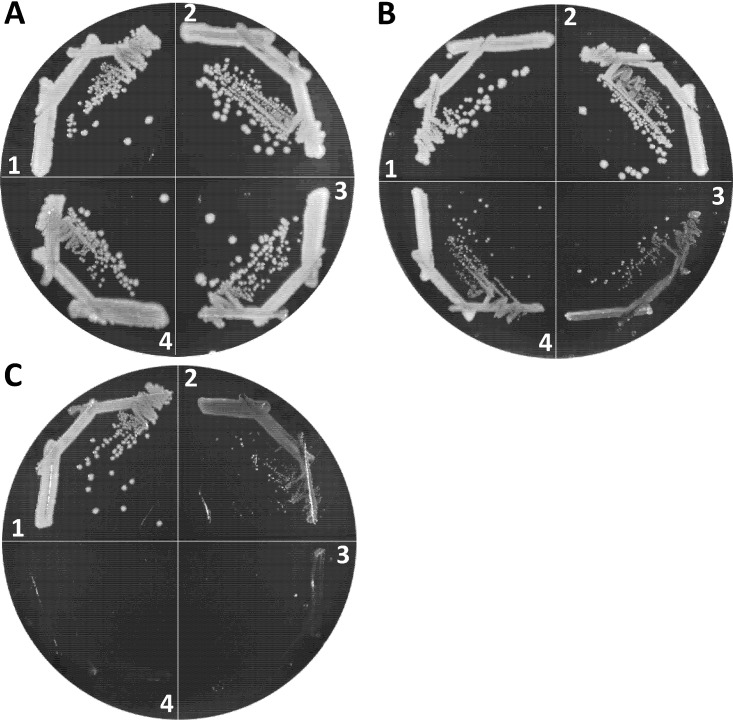
Effects of Δ*tonB* and Δ*aroB* mutations on bacterial growth under iron-replete and iron-depleted conditions. Bacterial growth on LBA plus 40 μM FeCl_3_ (A), LBA (B), and LBA plus 200 μM 2,2′-dipyridyl (C) was recorded after petri plates were incubated at 37°C for 24 h. Bacterial strains used: 1, RAM1292 (wild type); 2, RAM2553 (Δ*aroB*); 3, RAM2572 (Δ*tonB*); and 4, RAM2574 (Δ*aroB* Δ*tonB*).

We first determined the iron dependency of wild-type, Δ*aroB*, Δ*tonB*, and Δ*aroB* Δ*tonB* strains by growing them on Lysogeny broth agar (LBA), LBA supplemented with 40 μM FeCl_3_ and LBA containing 200 μM 2,2′-dipyridyl (DP), a synthetic iron chelator (Fig. 6). Bacterial growth in the absence of *aroB* was unaffected on LBA+FeCl_3_ or LBA (Fig. 6A and B). However, significant growth impairment of the Δ*aroB* strain occurred on LBA+DP plates (Fig. 6C), reflecting the loss of a major, enterobactin-mediated iron transport system. In contrast to Δ*aroB*, the deletion of *tonB* impaired bacterial growth even on LBA (Fig. 6B), which contains around 6 μM iron, and completely prevented growth on LBA+DP medium (Fig. 6C). The Δ*tonB* strain grew like WT on LBA+FeCl_3_, showing that the growth impairment of this strain on LBA was due to low accessibility to iron. Interestingly, growth of the Δ*aroB* Δ*tonB* double mutant improved slightly on LBA compared to the Δ*tonB* strain (Fig. 6B) but ceased again on LBA+DP (Fig. 6C). An improvement in growth of the double mutant compared to the Δ*tonB* strain on LBA may be due to the absence of extracellular enterobactin-Fe^3+^ complexes, which, when allowed to accumulate outside the Δ*tonB* cells, would sequester iron from the medium and further exacerbate growth defects (41). Because of the greater growth dependence of the Δ*tonB* and Δ*tonB* Δ*aroB* strains on external iron sources than the Δ*aroB* strain, we selected the former two genetic backgrounds to examine the effects of EnvZ/OmpR and porins in iron transport. It is worth noting that we did not determine bacterial growth rates by monitoring growth of liquid cultures because the Δ*tonB* strain frequently reverts without supplemented iron, and these faster-growing revertants take over the population to artificially display better-than-expected growth.

**FIG 7:**
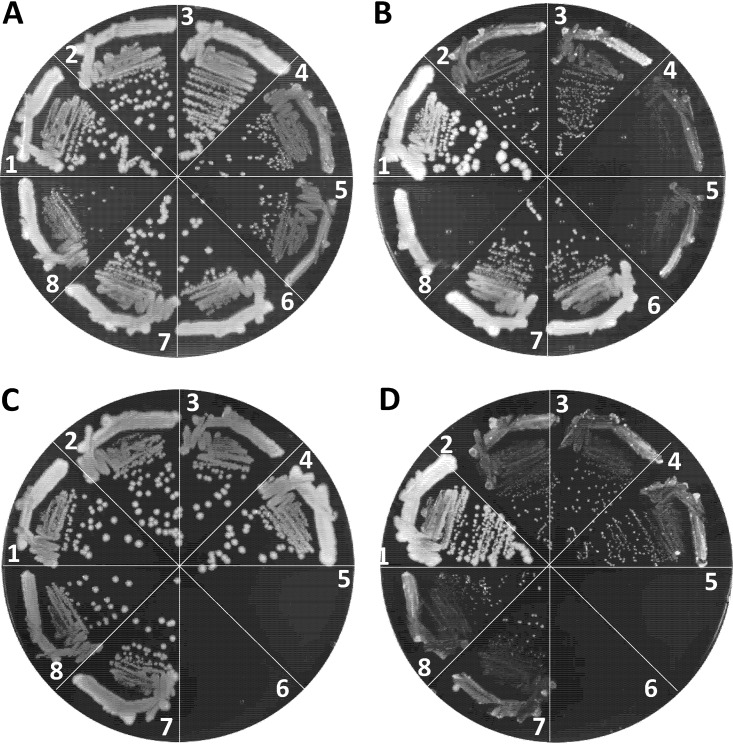
Effects of *ompR* and porin gene mutations on the growth of Δ*tonB* or Δ*tonB* Δ*aroB* mutants. Bacterial growth was monitored on LBA plus 40 μM FeCl_3_ (A and C) and LBA (B and D) after incubation of petri plates at 37°C for 24 h. Relevant genotypes of strains used in panels A and B: 1, RAM1292 (wild type); 2, RAM2572 (Δ*tonB*); 3, RAM2765 (Δ*tonB malPQ*::Tn*10*); 4, RAM2766 (Δ*tonB malPQ*::Tn*10 ompR101*); 5, RAM2767 (Δ*tonB* Δ*ompR*::Km^r^); 6, RAM2574 (Δ*tonB* Δ*aroB*::Km^r^); 7, RAM2771 (Δ*tonB* Δ*aroB*::Km^r^ *malPQ*::Tn*10*); and 8, RAM2772 (Δ*tonB* Δ*aroB*::Km^r^ *malPQ*::Tn*10 ompR101*). Relevant genotypes of strains used in panels C and D: 1, RAM1292 (wild type); 2, RAM2572 (Δ*tonB*); 3, RAM2769 (Δ*tonB* Δ*ompC*::Cm^r^); 4, RAM2768 (Δ*tonB* Δ*ompF*::Km^r^); 5 and 6, no bacteria; 7, RAM2792 (Δ*tonB* Δ*ompC*::Cm^r^ Δ*ompF*::Km^r^/p*ompC*); and 8, RAM2790 (Δ*tonB* Δ*ompF*::Km^r^ Δ*ompC*::Cm^r^/p*ompF*). p*ompF* and p*ompC* are pTrc99A plasmid clones expressing *ompF* and *ompC*, respectively. The expression of these plasmid-coded genes did not require induction by an inducer.

We employed two different null *ompR* alleles, *ompR101* and Δ*ompR*::Km^r^, both of which produce the OmpC^–^ OmpF^–^ phenotype. The *ompR101* allele was transduced into a Δ*tonB* background using a linked tetracycline resistance (Tc^r^) marker, *malPQ*::Tn*10*, while Δ*ompR*::Km^r^ was transduced directly using the Km^r^ gene that replaced the deleted *ompR* gene. Although both *ompR* alleles could be transduced in the Δ*tonB* strain when transductants were selected on LBA+FeCl_3_ containing appropriate antibiotics, the resulting null *ompR* Δ*tonB* transductants grew poorly compared to the *ompR*^+^ Δ*tonB* strain (Fig. 7A, sectors 4 and 5). In contrast, *ompR101* and Δ*ompR*::Km^r^ severely compromised growth of the Δ*tonB* strain on LBA not supplemented with FeCl_3_ (Fig. 7B, sectors 4 and 5). Similar to the Δ*tonB ompR101* strain, we were able to construct the Δ*aroB* Δ*tonB ompR101* strain on LBA+FeCl_3_ medium, where it grew poorly (Fig. 7A, sector 8) but not as poorly as on LBA without FeCl_3_, where the strain failed to form single colonies (Fig. 7B, sector 8). These observations pointed to a critical role for the EnvZ/OmpR TCS in iron transport in the absence of the high-affinity iron transport system.

Although the porin genes are the main targets of the EnvZ/OmpR regulatory system, transcription of other genes is also affected either directly or indirectly in the *ompR*-null mutant (26). Therefore, to establish unambiguously the importance of porins in iron transport, we attempted to delete the porin genes in a background devoid of the high-affinity transport system. In the Δ*tonB* background, the deletion of *ompC* or *ompF* individually did not significantly influence growth on LBA (Fig. 7C and D, compare sectors 3 and 4 to sector 2). Strikingly, however, we failed to delete *ompC* and *ompF* simultaneously, via P1 transduction of Δ*ompF*::Km^r^ and Δ*ompC*::Cm^r^ alleles, in the Δ*tonB* background even when transductants were selected on LBA+FeCl_3_ plates carrying appropriate antibiotics. In contrast, when the Δ*tonB* Δ*ompC* or Δ*tonB* Δ*ompF* double mutant was first complemented with a plasmid expressing one of the porin genes, the uncomplemented porin gene from the chromosome could be readily deleted by P1 transduction. The plasmid-complemented triple mutants displayed growth behavior similar to the uncomplemented Δ*tonB* Δ*ompC* and Δ*tonB* Δ*ompF* double mutants on LBA+FeCl_3_ or LBA (Fig. 7C and D, compare sectors 3 and 4 with sectors 7 and 8). It is worth noting that the Δ*tonB* Δ*ompC* and Δ*tonB* Δ*ompF* strains were not defective in P1 transduction, since drug resistant markers not associated with the porin genes or their regulators could be transduced readily into these strains. Moreover, unlike the Δ*tonB* strain, in the wild-type and Δ*aroB* backgrounds the *ompC* and *ompF* genes could be deleted simultaneously without causing iron dependency or a significant growth defect (Fig. S5). These data indicated for the first time that the OmpC and OmpF porins play a critical role in iron intake when the high-affinity iron transport system is blocked.

10.1128/mBio.01192-20.5 FIG S5 Effects of Δ*ompC* and Δ*ompF* gene mutations on the growth of wild-type and Δ*aroB* strains. Bacterial growth was monitored on an LBA plate incubated at 37°C for 24 h. The relevant genotypes are shown. Download FIG S5, TIF file, 0.5 MB. Copyright © 2020 Gerken et al. 2020 Gerken et al. This content is distributed under the terms of the Creative Commons Attribution 4.0 International license.

## DISCUSSION

Although the EnvZ/OmpR TCS is classically associated with the regulation of the OmpC and OmpF porins in response to medium osmolarity (23), recent transcriptomics and chromatin immunoprecipitation analyses showed that it is a global regulatory system (26, 37, 42). Indeed, missense alleles of *envZ*, called *perA* and *tpo*, isolated over 3 decades ago, were shown to also influence nonporin regulons, including *pho* and *mal* (43–45). A separate study revealed that *perA* lowered the expression of three iron-regulated proteins without an apparent reduction in the rate of enterobactin secretion (27). This led these authors to suggest that the effect of the *perA* (*envZ*) allele on the expression of iron-regulated proteins is most likely posttranscriptional (27).

**FIG 8:**
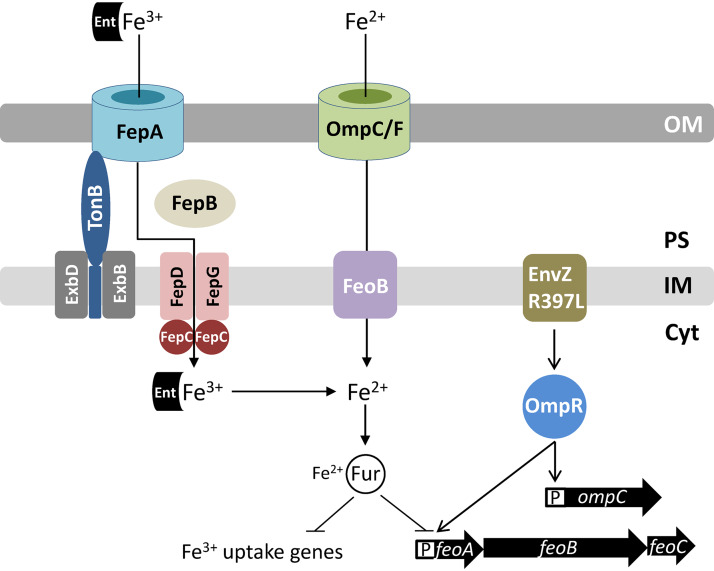
Diagram showing regulation of the ferric (Fe^3+^) and ferrous (Fe^2+^) uptake systems in E. coli. Fur-Fe^2+^ is the master regulator of transcription of genes involved in iron metabolism. Under aerobic growth conditions, where Fe^3+^ is the major source of iron, E. coli secretes enterobactin (Ent) in the medium to chelate Fe^3+^. The Fe^3+^-chelate complex is transported back into the cell through the outer membrane receptor protein, FepA. The TonB-ExbB-ExbD complex of the inner membrane facilitates FepA channel opening. In the periplasm, FepB interacts with the Fe^3+^-chelate and delivers it to the FepDGC complex for transport into the cytoplasm. Under microaerobic or anaerobic growth conditions, Fe^2+^ is the main source of iron. It is brought into the cell via porins OmpC and OmpF and FeoB. The EnvZ/OmpR two-component system, classically known for regulating the expression of the *ompC* and *ompF* porin genes, also induces *feo* expression when hyperactivated due to a specific mutation in *envZ* (*envZ_R397L_*). This positive effect of EnvZ_R397L_/OmpR on *feo* expression can overcome the negative effect of Fur-Fe^2+^ on *feo* expression, thus tipping the balance in favor of ferrous over ferric transport. Porins and EnvZ/OmpR play a crucial role in iron acquisition in a TonB-deficient background that lacks functional ferric transport systems. Abbreviations: OM, outer membrane; PS, periplasm; IM, inner membrane; Cyt, cytoplasm; P, promoter.

In the present study, we sought to resolve the mechanism by which the activated EnvZ/OmpR TCS reduces expression of genes involved in iron homeostasis and determine the role of porins in iron acquisition. We used the *envZ_R397L_* allele, which is phenotypically similar to the pleotropic *perA* and *tpo* alleles of *envZ*, i.e., in the *envZ_R397L_* background, OmpC levels go up, while those of OmpF and LamB go down dramatically (29). The RT-qPCR (this work) and the whole-genome microarray data (30; unpublished data) showed that in the presence of *envZ_R397L_* the transcript levels of several Fur-controlled genes, including *fecA*, *fepA*, *fhuA*, and *fhuF*, decreased significantly. In the case of *fhuA* and *fhuF*, the effects of *envZ_R397L_* required Fur, while expression of *fecA* and *fepA* was still reduced by *envZ_R397L_* in the absence of Fur. These observations indicated the involvement of at least two different mechanisms by which *envZ_R397L_* affected iron regulon. In support of the Fur-dependent mechanism, the whole-cell EPR data confirmed the presence of significantly elevated levels of accessible iron in the *envZ_R397L_* strain. Several observations supported the hypothesis that in the *envZ_R397L_* mutant, FeoB and OmpC are responsible for increased intracellular Fur-Fe^2+^ level (Fig. 8). First, unlike the expression of genes involved in ferric iron transport or metabolism, expression of the *feoAB* genes involved in ferrous iron transport went up dramatically in the *envZ_R397L_* background. This increase in the expression of the ferrous iron transport system had an adverse effect on the ferric iron transport system, since the absence of FeoB, the ferrous permease, abolishes or significantly reduces the negative effects of *envZ_R397L_* on ferric transport/metabolic genes. Second, like FeoB, the absence of OmpC (*envZ_R397L_* already severely represses *ompF* expression [29]) largely negated the inhibitory effects of *envZ_R397L_* on ferric transport/metabolic genes. Because the single deletion of *feoB* or *ompC* and the simultaneous deletion of *feoB* and *ompC* reversed the effects of *envZ_R397L_* on *fecA*, *fepA*, *fhuA*, and *fhuF* to the same extent, it indicated that FeoB and OmpC must act in the same pathway to transport ferrous iron into the cell and elevate Fur-Fe^2+^ levels. Third, the absence of FeoB or OmpC in an EnvZ^+^ background caused derepression of six Fur-controlled genes, indicating that the ferrous iron transport pathway is active under our experimental conditions and that *envZ_R397L_* enhances this pathway to achieve its inhibitory effects on the ferric transport system. Lastly, we provided direct evidence of excessive iron inside the *envZ_R397L_* mutant by whole-cell EPR spectroscopy measurements, which showed that, as in the Δ*fur* mutant, the level of accessible iron in the *envZ* mutant rose 4-fold over that present in the parental strain. Moreover, this increase in the intracellular free pool of iron in the *envZ_R397L_* mutant was dependent on FeoB. From these observations, we conclude that the upregulation of the OmpC-FeoB ferrous iron transport pathway by *envZ_R397L_* elevates the intracellular Fur-Fe^2+^ level, which, in turn, represses the expression of iron-regulated genes (Fig. 8). These effects of *envZ_R397L_* required functional OmpR since the presence of *ompR_D55Y_*, which confers a null phenotype, neutralized all *envZ_R397L_* phenotypes.

Whereas *envZ_R397L_*-mediated reduction in *fhuA* and *fhuF* transcript levels required Fur, the effects of *envZ_R397L_* on *fepA* and *fecA* transcripts did not. This suggested the existence of another regulatory mechanism responsible for the *envZ_R397L_*-mediated downregulation of *fepA* and *fecA* that did not involve Fur. Previous studies showed that the plasmid-mediated overexpression of OmrA and OmrB small RNAs, whose expression is under the EnvZ/OmpR control, can downregulate *fepA* and *fecA* transcript levels (31, 46). We have shown that *envZ_R397L_* increases OmrA expression almost 10-fold (29). This increase in OmrA expression could contribute to the downregulation of *fepA* and *fecA*. However, the fact that deleting *ompC* or *feoB* in a Fur^+^ background abolishes *envZ_R397L_*-mediated downregulation of the ferric iron transport genes suggests that *envZ_R397L_*-mediated increase in OmrA and OmrB levels contributes little, if any, to *fepA* and *fecA* repression. Consistent with this notion, we found that deletions of Δ*omrA* and Δ*omrB* failed to reverse the negative effect of *envZ_R397L_* on *fepA* or *fecA* (Fig. S2). We conclude that a mechanism independent of Fur and OmrA and OmrB must also exist for the *envZ_R397L_*-mediated downregulation of *fepA* and *fecA*. A direct role of EnvZ/OmpR has not been ruled out.

As stated above, unlike the ferric transport genes, expression of the ferrous transport genes *feoAB*, which are also under the control of Fur, went up in the *envZ_R397L_* background. At first glance, this appears inconsistent with the notion that an increase in the Fur-Fe^2+^ level by *envZ_R397L_* should also decrease *feoAB* expression. Our data suggest that *envZ_R397L_* overcomes the repressive effect of Fur-Fe^2+^ on *feoAB* by activating their expression. Moreover, because *feoAB* expression in the *envZ_R397L_* background increases dramatically without Fur, it shows that Fur-Fe^2+^ does repress *feoAB* in the *envZ_R397L_* background, but the positive effect of *envZ_R397L_* on *feoAB* expression overwhelms the negative effect of Fur on these genes. The EMSA data showed that purified OmpR binds to the *feo* regulatory region containing a putative OmpR binding site, indicating that OmpR directly activates *feoAB* expression. Interestingly, the whole-genome microarray data from an Δ*ompR* Δ*envZ* (porin-minus) strain showed that *feoB* transcript levels decreased 2-fold, whereas those of *fepA* and *fecA* increased 2-fold (26). These observations are consistent with our proposal that the EnvZ/OmpR TCS directly stimulates the FeoB-OmpC pathway to increase intracellular Fe^2+^ levels and thus active Fur-Fe^2+^ complexes, which then downregulate the expression of the ferric iron transport genes.

The proposed role of EnvZ/OmpR in iron homeostasis is similar to that suggested for RstA in *S.* Typhimurium (35). The authors found that overexpression of RstA increased *feoAB* expression and repressed *fhuA* and *fhuF* expression. A RstA binding site was identified in the *feo* promoter and the EMSA data confirmed that RstA bound there (35). The RstA binding motif “TACAtntngtTACA” resembles that of OmpR’s “GTTACAnnnnGTTACA” and not surprisingly, both proteins regulate overlapping genes by binding to the similar sequences (38). Our EMSA data showed that OmpR binds to the *feo* promoter region. Specifically, it binds to a DNA fragment containing the sequence “ttATCAtttcattAACA” located 278 bp upstream of the start codon of *feoA*. The OmpR binding studies carried out here involved the purified protein not modified by *in vitro* phosphorylation. Therefore, it is possible that stronger binding and/or additional binding sites may be discovered with phosphorylated OmpR. It is worth noting that in previous EMSAs, unphosphorylated RstA from *S.* Typhimurium and E. coli was shown to bind to their target promoter sequences (35, 47). Further work will be required to identify the exact binding sequences and to determine whether OmpR and RstA bind to the same, overlapping, or distinct regulatory sequences of the *feo* operon.

Our work also revealed for the first time the essential role of OmpC and OmpF porins in iron acquisition when the TonB-dependent ferric transport pathways are inoperative. The absence of OmpC or OmpF produced no growth defects in the Δ*tonB* background on LBA supplemented with iron, but the construction of a triple-knockout mutant (Δ*tonB* Δ*ompC* Δ*ompF*) required the expression of at least one of the porin genes from a plasmid replicon. Interestingly, unlike the porin-devoid triple-knockout mutant, we were able to construct Δ*tonB* Δ*ompR* and Δ*tonB* Δ*aroB* Δ*ompR* mutants, albeit only on LBA supplemented with iron. In the Δ*ompR* background, *ompC* and *ompF* porin expression is extremely low but presumably not zero, which is the case in the Δ*tonB* Δ*ompC* Δ*ompF* mutant. We think that this extremely low porin expression permits the construction of the Δ*tonB* Δ*ompR* strain, which can form single, albeit very small colonies, but only on LBA supplemented with iron. Whereas the diffusion of ferrous iron across the outer membrane occurs via OmpC or OmpF channels, at least three proteins—FeoB, MntH, and ZupT—can transport ferrous iron across the inner membrane of E. coli cells (48). Consistent with this, a Δ*tonB* Δ*feoB* double mutant is viable and grows like the Δ*tonB* mutant (data not shown).

Although the essential role of porins in iron acquisition becomes apparent without the TonB-dependent, high-affinity ferric iron transport systems, their derepression without OmpC or FeoB indicate that the porin-mediated iron transport is active even in the presence of the TonB-dependent high-affinity iron transport systems. The importance of the porin-FeoB pathway for bacterial growth should further increase as E. coli cells enter microaerobic or anaerobic environments where the ferrous species predominates. The involvement of porin and FeoB in iron-dependent growth and/or virulence has been reported for several bacteria, including E. coli (9), *S.* Typhimurium (10; 11), Helicobacter pylori (12), Vibrio cholerae (49), and Mycobacterium smegmatis (50). Interestingly, M. smegmatis porins increase ferric citrate uptake (50). Similarly, a study reported liganded iron uptake via the OprF porin in Pseudomonas aeruginosa (51). There are no definitive reports in E. coli showing the involvement of porins in liganded iron transport, even for ferric citrate, whose size is below the diffusion limits of the porins (52). Regardless of these ambiguities, published reports and the work carried out here highlight the importance of the porin/FeoB-mediated iron transport pathways in iron homeostasis.

## MATERIALS AND METHODS

### Bacterial strains, media, and chemicals.

Escherichia coli K-12 strains used in this study were constructed from MC4100 (53) and are listed in Table 1. Lysogeny broth (LB) was prepared using LB broth EZMix powder (Lennox). LB agar (LBA) medium contained LB plus 1.5% agar (Becton Dickinson). ONPG (2-*ortho*-nitrophenyl-β-d-galactopyranoside) was purchased from Acros. Diethylenetriaminepentaacetic acid (DTPA) and desferrioxamine were obtained from Sigma-Aldrich. All other chemicals were of analytical grade. The growth medium was supplemented with ampicillin (50 μg/ml), chloramphenicol (12.5 μg/ml), kanamycin (25 μg/ml), or tetracycline (10 μg/ml) when necessary. To induce plasmid-borne gene expression, 0.2% l-arabinose or 0.4 mM IPTG (isopropyl-β-d-thiogalactopyranoside) was added to the medium.

**TABLE 1:** Bacterial strains used in this study

Strain	Relevant genotype	Source or reference
RAM1292	MC4100 (Δ[*argF*-*lac*]*169* λ^–^ e14^–^ *flhD5301* Δ[*fruK*-*yeiR*]725 *relA1 rpsL150* (Str^r^) *rbsR22* Δ[*fimB*-*fimE*]*632 deoC1*) Δ*ara714*	61
RAM1541	RAM1292 *envZ_R397L_*	29
RAM2697	RAM1292 Δ*fur*::scar	This study
RAM2698	RAM1292 *envZ_R397L_* Δ*fur*::scar	This study
RAM2699	RAM1292 Δ*ompC*::scar	This study
RAM2700	RAM1292 *envZ_R397L_* Δ*ompC*::scar	This study
RAM2701	RAM1292 Δ*feoB*::scar	This study
RAM2702	RAM1292 *envZ_R397L_* Δ*feoB*::scar	This study
RAM2703	RAM1292 *envZ_R397L_* Δ*feoB*::scar Δ*ompC*::scar	This study
RAM2704	RAM1292 Δ*ompR*::scar	This study
RAM2705	RAM1292 Δ*ompR*::scar pBAD24-*ompR_6His_*	This study
RAM2707	RAM1292 Δ*ompR*::scar pBAD24	This study
RAM2708	RAM1292 pBAD24	This study
RAM2709	RAM1292 pBAD24-*ompR_6His_*	This study
RAM2711	RAM1292 Δ*feoA*::*lacZ*-Km^r^	This study
RAM2712	RAM1292 Δ*feoA*::*lacZ*-Km^r^ *envZ_R397L_*	This study
RAM2713	RAM1292 Δ*feoA*::*lacZ*-Km^r^ Δ*fur*::scar	This study
RAM2714	RAM1292 Δ*feoA*::*lacZ*-Km^r^ Δ*fur*::scar *envZ_R397L_*	This study
RAM2715	BL21(DE3) pET24d-*ompR_6His_*	This study
RAM2469	RAM1292 Δ*fepA*::*lacZ*-Km^r^	This study
RAM2470	RAM1292 Δ*fepA*::*lacZ*-Km^r^ *envZ_R397L_*	This study
RAM2471	RAM1292 Δ*fepA*::*lacZ*-Km^r^ Δ*fur*	This study
RAM2472	RAM1292 Δ*fepA*::*lacZ*-Km^r^ Δ*fur envZ_R397L_*	This study
RAM2473	RAM2469 Δ*ompC*::Cm^r^	This study
RAM2474	RAM2469 Δ*feoB*::Cm^r^	This study
RAM2475	RAM2470 Δ*ompC*::Cm^r^	This study
RAM2476	RAM2470 Δ*feoB*::Cm^r^	This study
RAM2477	RAM2469 Δ*ompF*::Cm^r^	This study
RAM2478	RAM2470 Δ*ompF*::Cm^r^	This study
RAM2505	RAM1292 Δ*tonB*::Km^r^	This study
RAM2553	RAM1292 Δ*aroB*::Km^r^	This study
RAM2572	RAM2505 Δ*tonB*::scar (via pCP20)	This study
RAM2574	RAM2572 Δ*aroB*::Km^r^	This study
RAM2623	RAM2553 Δ*aroB*::scar	This study
RAM2625	RAM2574 Δ*aroB*::scar (via pCP20)	This study
RAM2765	RAM2572 *malPQ*::Tn*10*-*ompR*^+^	This study
RAM2766	RAM2572 *malPQ*::Tn*10*-*ompR101*	This study
RAM2767	RAM2572 Δ*ompR*::Km^r^	This study
RAM2768	RAM2572 Δ*ompF*::Km^r^	This study
RAM2769	RAM2572 Δ*ompC*::Cm^r^	This study
RAM2771	RAM2574 *malPQ*::Tn*10*-*ompR*^+^	This study
RAM2772	RAM2574 *malPQ*::Tn*10*-*ompR101*	This study
RAM2790	RAM2768 (pTrc99A-*ompF*)	This study
RAM2792	RAM2769 (pTrc99A-*ompC*)	This study
RAM2795	RAM2790 Δ*ompC*::Cm^r^	This study
RAM2796	RAM2792 Δ*ompF*::Km^r^	This study
RAM2920	RAM2469 Cm^r^ linked to *envZ*^+^	This study
RAM2921	RAM2470 Cm^r^ linked to *envZ_R397L_*	This study
RAM2922	RAM2799 Cm^r^ linked to *ompR_D55Y_ envZ_R397L_*	This study
RAM2924	RAM2711 Cm^r^ linked to *envZ*^+^	This study
RAM2925	RAM2711 Cm^r^ linked to *envZ_R397L_*	This study
RAM2926	RAM2711 Cm^r^ linked to *ompR_D55Y_ envZ_R397L_*	This study
RAM2928	RAM2711 pBAD24 (Ap^r^)	This study
RAM2932	RAM2711 pBAD24-*ompR_6His_*	This study

### Genetic and DNA methods.

Standard bacterial genetic methods, including P1 transduction and plasmid transformation, were carried out as described by Silhavy et al. (54). To clone the *ompR* and *rstA* genes into pBAD24 (55) and pET24d(+) (Novagen), DNA corresponding to their open reading frames (ORFs) were amplified by PCR using primers that carried appropriate restriction enzyme sites for cloning. The reverse primers used for cloning into pBAD24 additionally contained nucleotides encoding six consecutive histidine codons. (Primer sequences are available upon request.) Deletion of the *fepA*, *feoA*, *feoB*, and *feoAB* genes from their chromosomal locations and subsequent scaring of the antibiotic-resistant marker at the deletion sites were done using the λ-Red-mediated gene recombination method (56). Deletions were confirmed by PCR and DNA sequence analyses. In some instances, promoterless *lacZY* genes were recombined at the deletion scar site by the method of Ellermeier et al. (57).

### RNA isolation, real-time quantitative PCR, and microarray analyses.

Total RNA was extracted from 5 ml cells grown to log phase (OD_600_ ∼0.6) at 37°C using TRIzol Max bacterial RNA isolation kit (Invitrogen). RNA was further purified using the RNeasy kit (Qiagen), and the quality of RNA was assessed by using an Agilent 2100 bioanalyzer (Agilent Technologies). The purified RNA was then converted to either single-stranded cDNA for use in RT-qPCR or double-stranded cDNA for use in DNA microarray analysis.

For RT-qPCR, single-stranded cDNA was synthesized from 10 μg of RNA using 100 pM random hexamer primer (Integrated DNA Technologies) and M-MuLV reverse transcriptase (New England Biolabs). After reverse transcription, cDNA was treated with 5 U of RNase H (New England Biolabs) for 20 min at 37°C, followed by purification with a QIAquick PCR purification kit (Qiagen). To quantify the RNA transcripts, 300 nM concentrations of primer specific to the gene of interest and 20 ng of cDNA was added to SYBR green PCR master mix (Applied Biosystems) in a 20-μl reaction. Primers were designed according to the manufacturer’s protocol included with the SYBR green PCR master mix and RT-qPCR reagents. Critical threshold (*C_T_*) values were determined by using an ABI Prism 7900HT sequence detection system (Applied Biosystems). The relative quantification of target transcripts was calculated according to the 2^–ΔΔ^*^CT^* method (58) using *ftsL* and *purC* as the endogenous control genes. Briefly, changes in *C_T_* value (Δ*C_T_*) for the gene of interest were calculated by subtracting that gene’s average *C_T_* from the average *C_T_* for the endogenous control gene. The Δ*C_T_* for the mutant was then subtracted from the wild-type strain’s Δ*C_T_* value to give the ΔΔ*C_T_* value. Each PCR was performed in triplicate and fold changes in transcript levels, along with the standard deviations, were calculated from at least two experiments (*n* ≥ 2).

For microarray analysis, an Invitrogen superscript double-stranded cDNA synthesis kit was used to generate double-stranded cDNA according to the manufacturer’s instructions. Single-stranded cDNA was synthesized from 10 μg of RNA using a 100 pM concentration of random hexamer primer (Integrated DNA Technologies) and Superscript II reverse transcriptase. Second strand synthesis was performed according to the manufacturer’s instructions, and the reaction was stopped with 0.5 M EDTA. RNA was then digested using RNase A (25 μg/ml final concentration), followed by treatment with phenol-chloroform and precipitation with ethanol. Double-stranded cDNA was further purified with a QIAquick PCR purification kit (Qiagen) and quality tested by using an Agilent bioanalyzer. Cy3 fluorescently labeled cDNA was used to probe array slides printed with 4,254 E. coli ORFs. Array slides contained 8 probes per gene (in duplicate) corresponding to roughly 72,000 probes per sample. Sample labeling with Cy3 fluorescent dye, hybridization to the 4-plex array (0771112 E. coli K-12 EXP X4, catalog number A6697-00-01), washing, and one-color scanning were performed by Roche Nimblegen in accordance with their standard protocol. Analysis of gene expression profiles was performed using ArrayStar 2.0 software (DNAStar) with a focus on genes with a ≥2-fold change in gene expression. *P* values were generated with the Student *t* test, and false positives were minimized using false discovery rate analysis (59).

### Enzymatic assays.

A β-galactosidase assay was performed according to the Miller method (60). Assays were carried out with at least two independent cultures. The β-galactosidase activity was expressed as Miller units (60). In some instances, kinetic analysis of enzyme activity was carried out using a VersaMax (Molecular Dynamics) microtiter plate reader in quadruplicate, and the activity was measured as the rate of ONPG cleavage divided by the cell density in each well.

### Electron paramagnetic resonance spectroscopy.

Free iron concentration in whole cells was determined by EPR spectroscopic analysis (32) with some modifications. Briefly, overnight grown bacterial cultures were diluted 1:100 in 200 ml of LB and grown shaking at 37°C until the optical density at 600 nm (OD_600_) reached 0.8. Cells were pelleted by centrifugation in a GSA rotor (Sorvall) for 10 min at 6,000 × *g*. Pellets were resuspended in 10 ml of LB containing 10 mM DTPA (to chelate extracellular iron) and 20 mM desferrioxamine (to chelate intracellular free or accessible ferric iron) and incubated with shaking for 37°C for 15 min. Cells were pelleted as described above and washed twice with 5 ml of ice-cold 20 mM Tris-HCl (pH 7.4). The final cell pellet was resuspended in 0.3 ml of ice-cold 20 mM Tris-HCl (pH 7.4) containing 30% glycerol. A 250-μl aliquot of this cell suspension was placed in a quartz EPR tube (length, 250 mm; external diameter, 4 mm [Wilmad-Labglass]). Tubes were frozen in loosely packed dry ice and then transferred to –80°C until the EPR analysis. The remaining cells were diluted 10^3^-fold to determine the OD_600_. Iron standards were prepared from a freshly prepared 10 mM FeCl_3_⋅6H_2_O stock in a buffer containing 20 mM Tris-HCl (pH 7.4) and 1 mM desferrioxamine. Theoretical concentrations of iron standards were 100, 50, 25, 10, 5, and 0 μM. The actual iron concentrations were determined by measuring the OD_420_ of each standard and using the formula following: molar concentration = *A*_420_/ε, where [Fe]ε is 2.865 mM^−1 ^Cm^−1^. A 250-μl aliquot of each standard was placed in separate EPR tubes that were then frozen. The standard curve was generated by plotting EPR signals against actual iron concentrations (Fig. S6). The free iron concentration for each strain was determined from the EPR data and the standard curve. The intracellular free iron concentration was then deduced by integrating the intracellular volume of the cell (1 ml of 1.0 OD_600_ cells has an intracellular volume of 0.00052 ml; Jim Imlay, unpublished data) and using the following formula: intracellular free iron concentration = [Fe] from standard curve/cell paste OD_600_ × 0.00052 ml.

10.1128/mBio.01192-20.6 FIG S6 Standard curve of ferric chloride solutions of known concentrations. The actual concentrations of ferric chloride solutions were determined by measuring the absorbance at 420 nm and then utilizing the following formula: molar concentration = *A*_420_/ε, where [Fe]ε is 2.865 mM^−1^ Cm^−1^. These values were plotted against the values obtained for the same solutions by EPR spectroscopy. We used peak-to-peak EPR measurements instead of the double integration values due to a greater confidence of the former at lower ferric chloride concentrations. The standard curve was used to measure iron concentrations in the whole cell. Download FIG S6, TIF file, 0.5 MB. Copyright © 2020 Gerken et al. 2020 Gerken et al. This content is distributed under the terms of the Creative Commons Attribution 4.0 International license.

EPR measurements were carried out at the EPR Facility at Arizona State University. Continuous wave EPR spectra were recorded using an ELEXSYS E580 CW X-band spectrometer (Bruker, Rheinstetten, Germany) equipped with a model 900 EPL liquid helium cryostat (Oxford Instruments, Oxfordshire, UK). For all measurements, the magnetic field modulation frequency was 100 kHz, the amplitude was 1.25 mT, the microwave power was 10 mW, the microwave frequency was 9.44 GHz, the sweep time was 42 s, and the temperature was 20 K.

### OmpR purification.

OmpR was purified from BL21(DE3) cultures carrying a pET24-*ompR_6His_* plasmid. Overnight cultures, grown without IPTG, were diluted 1:100 in 1 liter of LB, grown with vigorous shaking for 90 min, and then supplemented with IPTG and grown for another 2 h. The cells were pelleted, washed with 10 mM Tris-HCl (pH 7.5), resuspended in lysis buffer (10 mM Tris-HCl [pH 7.5], 1 mM EDTA, 100 μg/ml lysozyme), and incubated on ice for 30 min. MgCl_2_ (10 mM final), phenylmethylsulfonyl fluoride (2 mM final), and DNase I (40 μg/ml final) were then added to the cell suspension. Cells were lysed by passage through a French pressure cell three times, and the lysate was centrifuged at low speed to remove unlysed cells. Envelopes were removed from the lysate by ultracentrifugation at 105,000 × *g* for an hour at 4°C. Supernatant was filtered through a 0.45-μm syringe filter, and the filtrate was subjected to nickel affinity column chromatography using buffers for protein binding (20 mM sodium phosphate [pH 7.4], 20 mM imidazole, and 50 mM NaCl), washing (20 mM sodium phosphate [pH 7.4], 50 mM imidazole, and 300 mM NaCl), and elution (20 mM sodium phosphate [pH 7.4], 300 mM imidazole, and 300 mM NaCl). Samples from eluted fractions were analyzed by SDS-PAGE, and protein bands were visualized after Coomassie blue staining (Fig. S3). Fractions representing OmpR_6His_ peaks were pooled and dialyzed against a buffer containing 20 mM sodium phosphate (pH 7.4) and 300 mM NaCl. Purified proteins were stored at 4°C in the dialysis buffer supplemented with glycerol (5% final concentration), EDTA (0.1 mM, final concentration), and dithiothreitol (0.1 mM, final concentration).

### Electrophoretic mobility gel shift assays.

EMSAs were carried out using a LightShift chemiluminescent EMSA kit (Thermo Scientific). *ompC* and *feoABC* promoter fragments were generated by PCR using primers specific to the region of interest, with one of the primers biotinylated. Biotin-labeled DNA probes (20 fmol), purified OmpR_6His_ (100 pmol), and other relevant reagents provided with the kit were incubated for 20 min at room temperature, and the reaction was stopped by adding 5× loading buffer. The mixture was analyzed by 5% acrylamide gel electroblotted onto polyvinylidene difluoride Immobilon-P membrane (Millipore) using a Mini Trans-Blot cell (Bio-Rad). After transfer, DNA was cross-linked to the membrane using Hoefer UV Crosslinker and incubated with stabilized streptavidin-HRP conjugate for an hour. DNA was detected by a molecular imager ChemiDoc XRS system (Bio-Rad) after the membrane was incubated for 5 min with freshly mixed luminol/enhancer and stable peroxide solutions.
