# Supplementary figures and images for: Roles of the EnvZ/OmpR Two-Component System and Porins in Iron Acquisition in Escherichia coli
Source: mBio. 2020 Jun 23;11(3):e01192-20. doi: 10.1128/mBio.01192-20 (PMC7315122; doi:10.1128/mBio.01192-20)

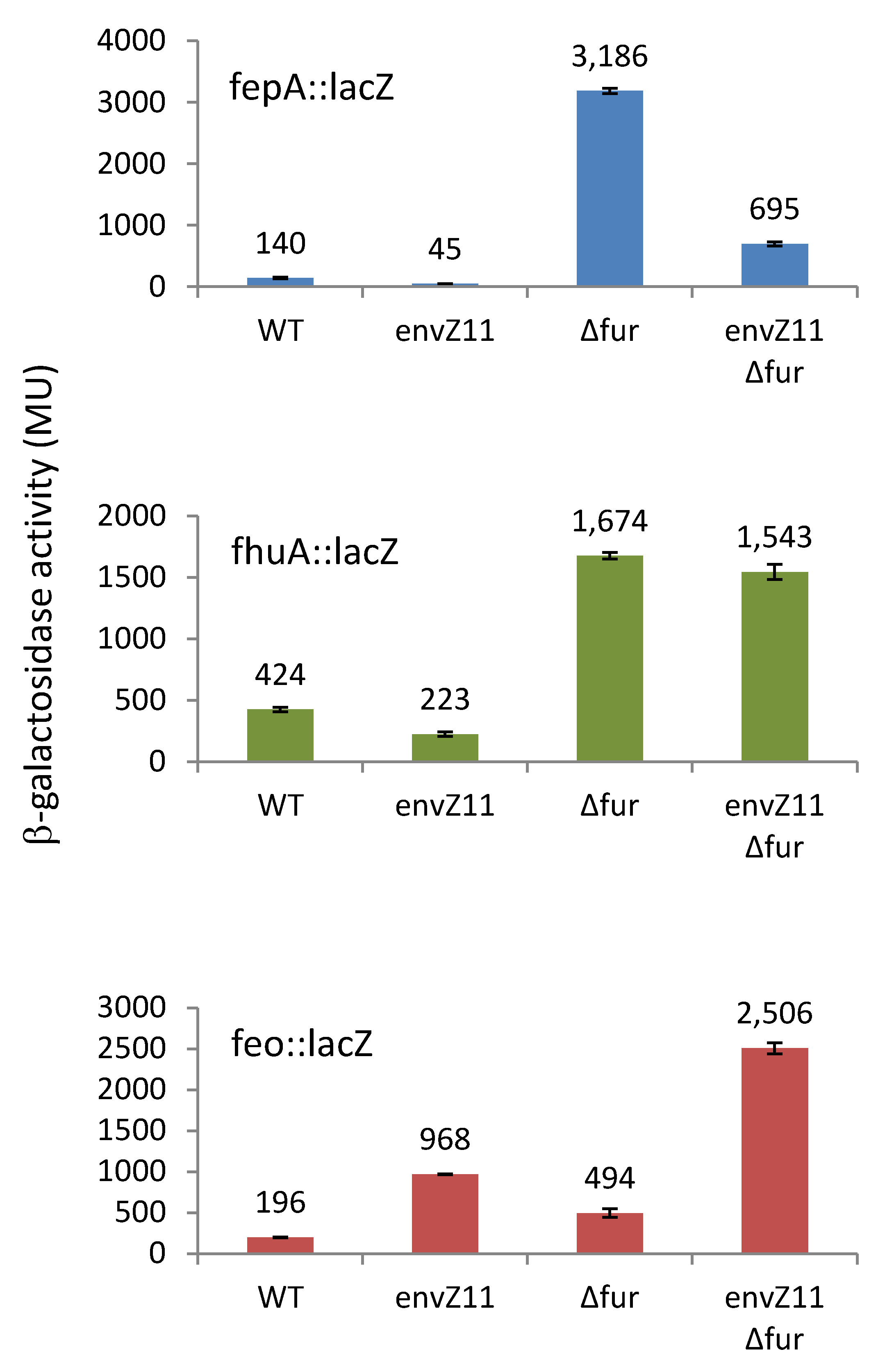

Supplement: FIG S1 [file mBio.01192-20-sf001.tif]

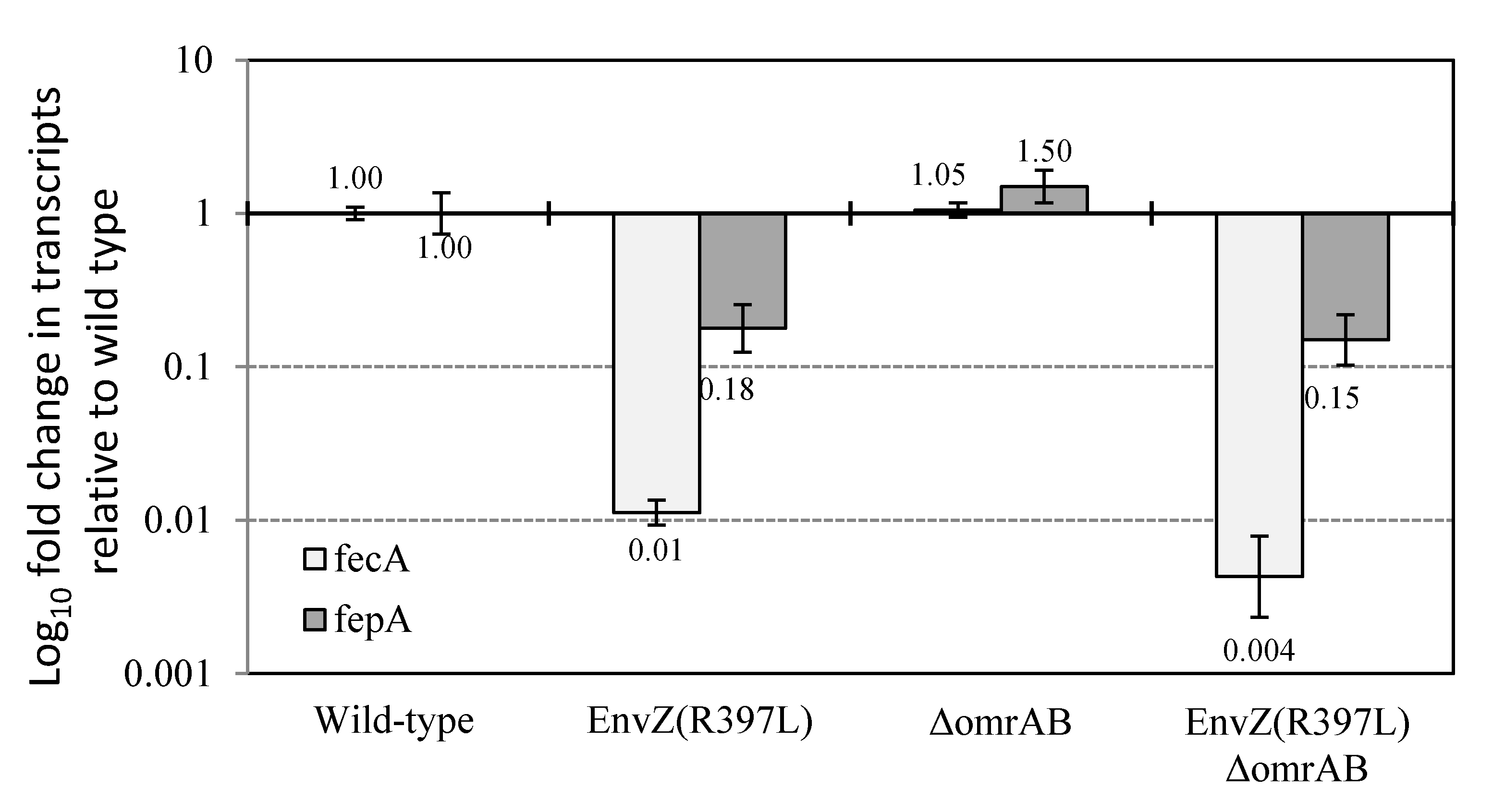

Supplement: FIG S2 [file mBio.01192-20-sf002.tif]

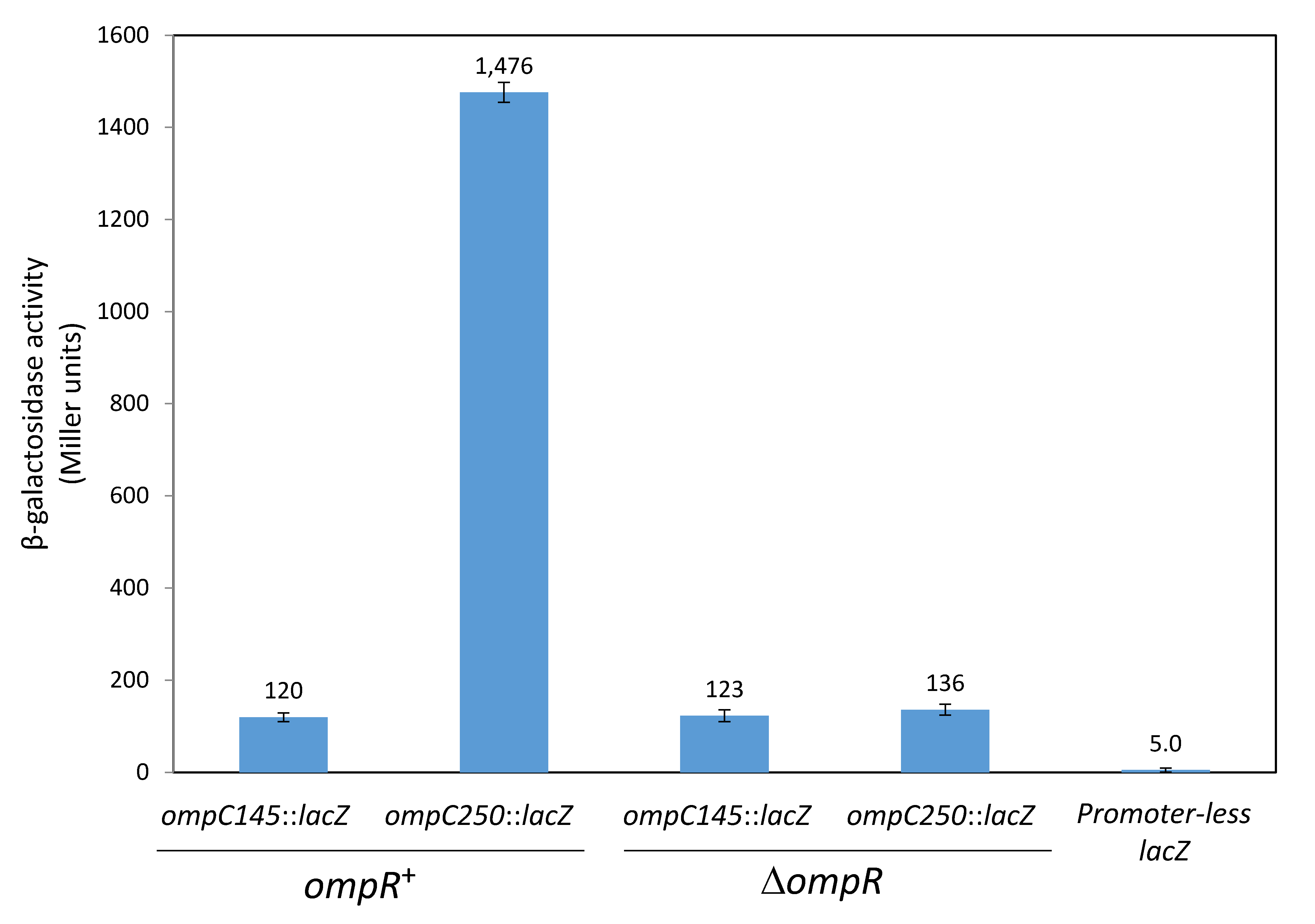

Supplement: FIG S4 [file mBio.01192-20-sf004.tif]

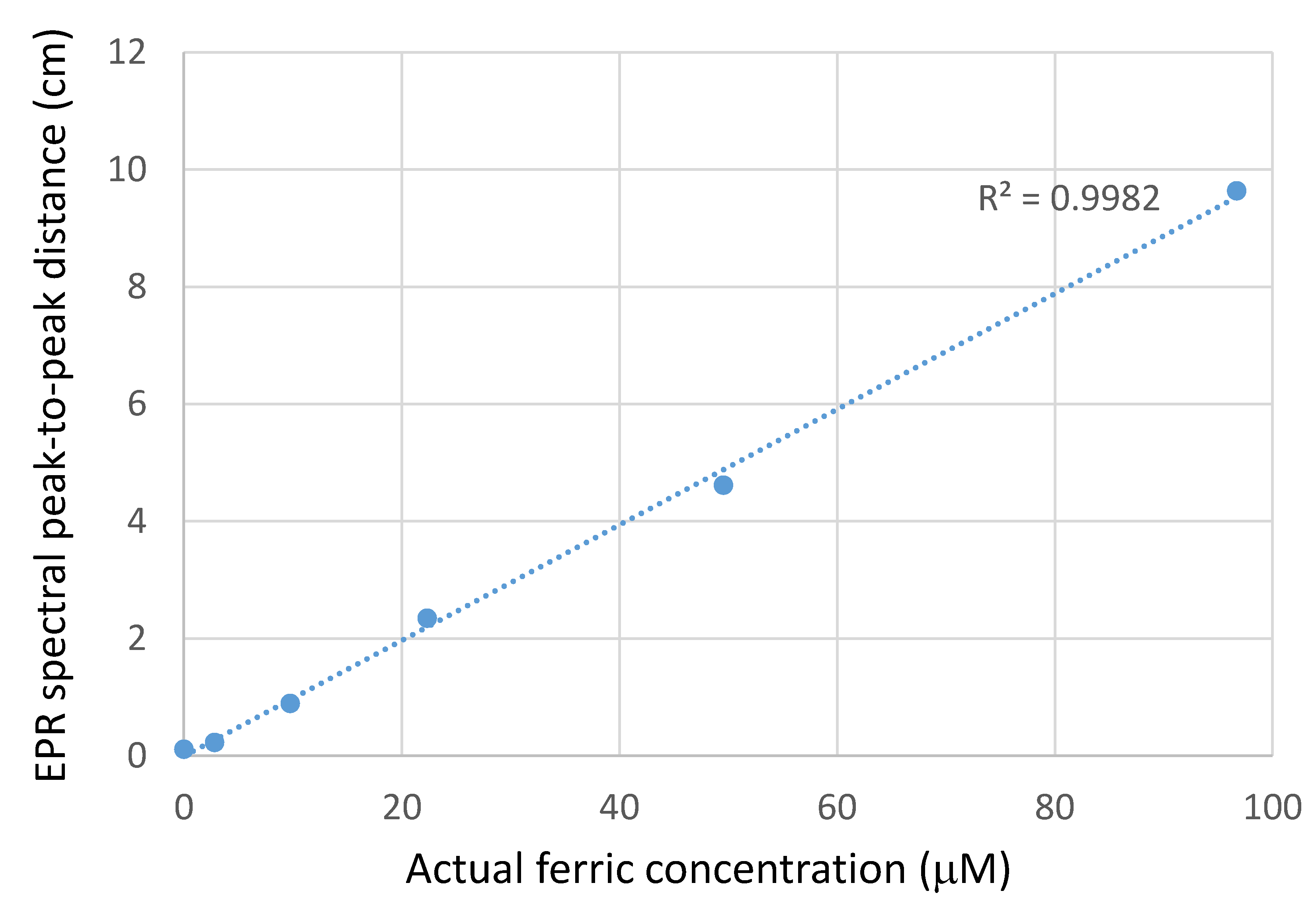

Supplement: FIG S6 [file mBio.01192-20-sf006.tif]
